# Usefulness of direct intratumoral administration of doxorubicin hydrochloride with an electro-osmosis–assisted pump

**DOI:** 10.3389/fddev.2023.1150894

**Published:** 2023-04-04

**Authors:** Ayu Ito, Shoko Itakura, Yuya Hasegawa, Miyu Hashimoto, Akie Okada, Mamoru Hirafuji, Hidenori Nakamura, Kenji Sugibayashi, Hiroaki Todo

**Affiliations:** ^1^ Faculty of Pharmacy and Pharmaceutical Sciences, Josai University, Saitama, Japan; ^2^ atDose Co., Ltd., Yokohama, Kanagawa, Japan; ^3^ Faculty of Pharmaceutical Sciences, Josai International University, Togane, Chiba-ken, Japan

**Keywords:** electro-osmotic flow pump, micro-pumping systems, anti-tumor effect, direct administration, intratumor administration

## Abstract

Patients receiving chemotherapy by intravenous (*i.v.*) or oral administration of anticancer drugs often experience side effects. In this study, an electro-osmotic flow (EO) pump was used for the direct administration of an anticancer drug with minimum side effects. Doxorubicin hydrochloride (DXR) was used as an anticancer drug, and its antitumor effect and toxicity were evaluated in comparison with *i.v.* administration. Balb/c female mice were subcutaneously transplanted with a breast cancer cell line (4T1/Luc) stably expressing luciferase, and 20 μL of DXR solution (1.0 mg/mL) was administered intratumorally (*i.t.*) at a slow rate (0.6 µL/min) using an EO pump or rapidly using a syringe. For comparison, 100 μL of DXR solution was injected through the tail vein at the same dose and a 5-times higher dose. A tumor growth inhibitory effect without significant weight loss was observed with direct *i.t.* administration of DXR using an EO pump. On the other hand, no suppressive tumor growth effect was observed with *i.v.* administration of DXR at the same dose. Although there was no significant difference in the suppression effect on tumor growth between *i.t.* administration with EO pump and syringe, the peripheral skin concentration of DXR were decreased after slow administration with EO pump compared with that after rapidly administration with a syringe. These results indicated that direct *i.t.* administration of DXR with lower dosing using an EO pump at slower administration rate may be useful for exhibiting antitumor effects and suppressing systemic side effects. In addition, the blood concentration and the peripheral skin concentration of DXR after administration at lower rate with EO pump were decreased compared with those after the rapidly administration with a syringe.

## Introduction

According to estimates of cancer incidence and mortality rates reported by the Global Cancer Observatory, 19.3 million new cancer cases and approximately 10 million cancer deaths are estimated worldwide by 2020 (Sung et al., 2021). Various treatment modalities have been developed, including surgery, radiation therapy, and chemotherapy, and combinations of these therapies are also being used. The main approach to solid tumor cancers is surgical removal. However, tumor recurrence and tumor metastasis after surgery lead to decreased quality of life (QOL) and increased mortality, which are issues that need to be addressed. To prevent tumor recurrence and reduce distant metastasis, applicable approaches should be performed to eradicate circulating tumor cells and residual tumors at the surgical site. In recent years, the utilization of immunotherapy and chemotherapy based on drug delivery systems (DDSs) after postoperative treatment of cancer has been receiving attention (Aquib et al., 2020). For chemotherapy, non-selective drug distribution causes severe unintended and undesirable side effects, resulting in many cancer patients suffering decreased QOL and reducing the efficacy of anticancer drugs. Drug delivery using nanoparticles *via* the enhanced permeability and retention (EPR) effect (Matsumura and Maeda, 1986) is a common strategy for anticancer chemotherapy for the development of therapeutic strategies with high tumor selectivity. If it is possible to inject the drug directly into tumor tissue with minimum drug distribution to normal tissue, this technique may provide another strategy to reduce side effects. The PLGA-based doxorubicin-loaded implants for tumor therapy has been reported. The implantation of PLGA formulation improved the efficacy of anti-cancer drug and has low toxicity to normal tissues (He et al., 2022).

Microelectromechanical systems technology-based DDSs are able not only to reduce the weight and the size of the device itself compared with conventional devices but also provide accurate and reliable delivery that can administer minute volumes of drug solutions. Various micro-pumping systems have already been reported, including piezoelectric (piezo) actuators (Liu et al., 2010), shape memory alloy actuators (Zainal et al., 2015), magnetic actuators (Rusli et al., 2018), rotary pump actuators (Pankhurst and Abdollahi, 2016), electrostatic actuators (Teymoori and Abbaspour-Sani, 2005), and electro-osmotic flow actuators (EOs) (Snyder et al., 2013), etc. Among these, EOs have advantages such as they are inexpensive to produce, the flow rate can be adjusted according to the loaded voltage, solution-pumping with high pressure and no pulsation is obtained with low electricity consumption, and it is possible to downsize the device.

EOs have been developed based on electro-osmosis, which is the movement of an uncharged liquid relative to a stationary charged surface due to an externally applied electric field (Bockris and Reddy, 1970). Silanol groups (Si-OH) are present on the surface of the inner wall of a glass capillary. When the pH of an aqueous solution is near neutral, free hydrogen ions (H^+^) are attracted to the silanol groups (formation of an electric double layer). When both ends of the capillary are immersed in the aqueous solution and electricity is applied, the hydrogen ions (H^+^) that are concentrated near the wall move toward the cathode electrode, and the entire solution flows in the cathode direction accordingly, which becomes an electro-osmotic flow (Hiroike, 2004).

A DDS for the treatment of diabetic macular edema with an implantable micropump has been reported (Humayun et al., 2014). In addition, continuous subcutaneous insulin infusion with a pump resulted in a greater reduction in glycated hemoglobin without a higher rate of hypoglycemia compared with multiple daily insulin injections (Bußmann et al., 2021). Furthermore, it has been reported that continuous administration of drugs with the use of chronomodulation infusion pumps provided therapeutic effects while minimizing side effects (Youan, 2004; Lévi et al., 2007). In the present study, doxorubicin hydrochloride (DXR) was selected as a model anticancer drug, and the antitumor effects and toxicity were investigated when direct intratumoral (*i.t.*) administration of DXR was conducted at a low dose and slow rate with an EO pump compared with its intravenous (*i.v.*) bolus injection and *i.t.* administration with a rapid injection rate.

## Materials and methods

### Materials

A stable luciferase-expressing breast cancer cell line (4T1/luc) was purchased from JCRB Cell Bank (National Institutes of Biomedical Innovation, Health and Nutrition, Osaka, Japan). Trypsin-EDTA, RPM-1640, and RIPA buffer were purchased from Fujifilm Wako Pure Chemicals Co., Ltd. (Osaka, Japan). Fetal bovine serum (FBS) was purchased from Sigma-Aldrich (St. Louis, MO, United States). DXR and daunorubicin hydrochloride (DAU) were purchased from Tokyo Kasei Kogyo Co., Ltd. (Tokyo, Japan). VivoGloTM Luciferin, *in vivo* grade, was purchased from Promega (Madison, WI, United States). An EO pump was kindly provided by atdose Corporation (Kanagawa, Japan).

### Preparation of tumor-bearing mice

Female BALB/c mice (weight 15–20 g, 6–8 weeks old) were purchased from Sankyo Lab Service Co., Ltd. (Tokyo, Japan) and kept in a room controlled at 25°C ± 2°C with a 12-hourly light/dark cycle (on-off time: 9:00–21:00). The mice were allowed free access to MF pellets (Oriental Yeast Co., Ltd., Tokyo, Japan) and water. All procedures were approved by the Josai University Animal Care and Use Committee (Sakado, Saitama, Japan) and complied with the National Institutes of Health’s Guide for the Care and Use of Laboratory Animals. After approval by the Josai University Ethics Committee (approved number: JU21007), the experimental animals were used in accordance with the Josai University Laboratory Animal Regulations. A suspension of 4T1/Luc cells in PBS was mixed with GeltrexTM (Thermo Fisher Scientific, MA, United States) at a final density of 3.3 × 10^7^ cells/mL. The mice were implanted subcutaneously with 30 µL of suspension into the dorsal side of both lower limbs under anesthesia with a triple anesthetic mixture (medetomidine hydrochloride: 0.15 mg/kg midazolam: 2 mg/kg butorphanol tartrate: 2.5 mg/kg). Effect of direct *i.t.* administration of DXR with the EO pump was evaluated after the tumor volume achieved around 100–150 mm^3^. As the comparator, rapid direct *i.t.* administration of DXR with a syringe and *i.v.* administration from tail vein were performed.

### DXR administration

**FIGURE 1:**
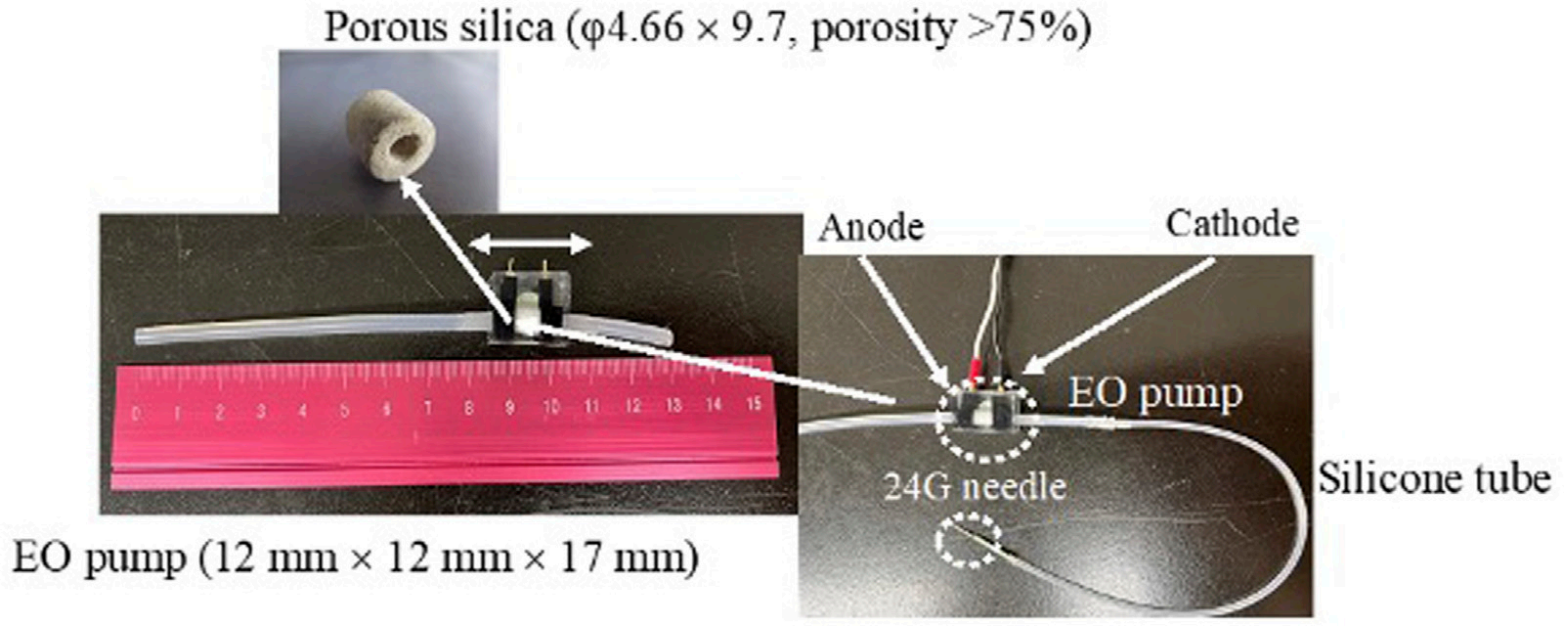
Set-up of the EO pump system and electro-osmotic flow in silica capillary. Filling water in a silica capillary has an electric double layer at the liquid-solid interface. External application of an electrical potential along the length of the capillary results in motion toward the cathode of some of the H+ ions in the liquid phase of the electric double layer. As they move under the influence of the applied field, they draw the liquid along them.

Because the flow rate and pressure of the electro-osmotic flow are related to the surface area of the capillary inner wall, it is necessary to equip a glass capillary with a porous structure to fabricate a small but high-performance EO pump (Nakamura, 2020). In the present study, the EO pump incorporated porous silica (φ4.66 × 9.7, porosity >75%) within a 12 mm × 12 mm × 17 mm (Figure 1) was used. Ultrapure water filled the EO pump to prevent air from entering, and then the EO pump was connected with a silicone tube (inner diameter 1 mm) that filled with 20 µL of 1.0 mg/mL DXR solution [prepared with pH 7.4 phosphate-buffered saline (PBS); the amount of DXR administered was 20 µg]. A 24G needle was connected to the end of the connected silicone tube. The feeding pressure of the aqueous solution by the EO pump was 25 kPa at 12 V load condition, and the power consumption was 18 µW. The *i.t.* administration rate of the filled solution into the silicone tube by the EO pump was 0.6 µL/min when a 24G needle was inserted into the tumor.

As the comparator, 0.2 mg/mL and 1.0 mg/mL of DXR solution were prepared with PBS, and 100 µL of 0.2 mg/mL or 1.0 mg/mL DXR solutions was administered by *i.v.* injection into the tail vein (the amount of DXR administrated was 100 µg). In addition, rapid *i.t.* administration of 20 µL of 1.0 mg/mL DXR solution was conducted with a syringe (within 5 s to the end of administration). PBS was used as a control formulation. DXR administration was conducted every 2 days. Tumor size, change in body weight, and luciferase expression were measured using an *in vivo* imaging system (IVIS) (PerkinElmer^®^ Ltd., Waltham, MA, United States) over 14 days. Direct *i.t.* administration of PBS and DXR using an EO pump and a syringe was carried out into the left lower extremity and the right lower extremity. The tip of the needle was injected into the center of the tumor.

### Evaluation of tumor growth

Tumor growth was evaluated using tumor volume and *in vivo* luminescence imaging. Tumor volumes were calculated using the formula:

$$Tumor\;volume=\left(length\right)\times {\left(width\right)}^{2}\times 0.5$$

Luminescent images of implanted 4T1/Luc luciferase were obtained using IVIS spectrum (PerkinElmer^®^ Ltd., Waltham, MA, United States). VivoGlo Luciferin was dissolved with PBS and injected intraperitoneally at 150 mg/kg 20 min before image acquisition. Luminescence images were acquired using an open filter during anesthesia with isoflurane. The radiance of the region of interest was determined using Living image^®^ (PerkinElmer^®^ Ltd., Waltham, MA, United States). In addition, the change in body weight ratio was calculated by dividing the body weight at 0–14 days with the body weight at 0 days to evaluate the systemic toxicity.

### Visual observation of DXR distribution in the peripheral part of the tumor after *i.t.* administration

The peripheral area of the tumor was visually observed just after finishing *i.t.* administration of DXR with syringe and EO pump. Changes observed visually were photographed with a digital camera.

### Quantification of DXR in tumor and skin around the tumor

4T1/Luc cell-bearing mice were prepared in accordance with the aforementioned method. When the tumor volume reached 100–150 mm^3^, the DXR solution was injected into the tumor using an EO pump or syringe. The tumor tissue and a 5 mm-wide region of normal skin tissue adjacent to the tumor (hereinafter, peripheral tissue) were excised 60, 120, and 180 min after finishing administration, and 20 µL of 1 mg/mL DAU as an internal standard (IS) and 1.2 mL of RIPA buffer were added to the excised skin. The tissue was homogenized using a POLYTRON^®^ PT 1200E (KINEMATICA, Switzerland) and ultrasonic homogenizer VCX-750 for 1 min (SONICS & MATERIALS, INC., United States).

Next, 1.2 mL of chloroform and 3 mL of methanol were added to the homogenate and mixed with a vortexer. After incubation at room temperature for 30 min, 1.5 mL of chloroform and 1.5 mL of distilled water were added to the mixture, followed by centrifugation at 760 × g for 5 min. The chloroform layer insoluble in a methanol/water was isolated and dried using nitrogen gas, then the residue was resuspended in PBS. The suspension was pretreated with acetonitrile and passed through a 0.45 µm filter. The quantity of DXR was quantified using a high-performance liquid chromatography (HPLC) system (Shimazu Co., Kyoto, Japan) equipped with a UV detector. A 20 µL aliquot of the sample solution was injected into a C18 column (Inertsil ODS-3, 5 μm, 250 mm × 4.6 mm I.D.; GL Sciences, Tokyo, Japan) with a flow rate of 1 mL/min and monitored at 480 nm. The sample was eluted using a mobile phase of 0.3% sodium dodecyl sulfate and 0.14% phosphoric acid in acetonitrile/water (1:1; vol/vol). The amount of DXR in each tissue was calculated using the peak area ratio, dividing the area of the peak obtained for the IS sample. Extraction procedure from tissues and detection condition of DXR were referred to the previous report (Yokoe et al., 2008) with some modifications.

### Quantification of DXR in plasma

After DXR solution was injected into tumor by using an EO pump or syringe, blood was collected from the orbit of the eye with heparin coated hematocrit tube under anesthesia at indicated time. The plasma was obtained by centrifugation of blood at 1,170 × *g* for 10 min at 4°C. 20 µL of plasma was mixed with 180 µL of acetonitrile and 6 µL of IS, followed by centrifugation of blood at 21,500 × *g* for 5 min at 4°C. The DXR of supernatant was quantified using a HPLC system according to the method described above.

### Statistical analysis

Statical analysis was performed using Dunnett’s test with JMP Pro version 16.0.0 (SAS Institute, Cary, NC, United States). *p*-values were considered statistically significant at *p* < 0.05 for tumor growth and for weight loss ratio.

## Results

### Visible observation of DXR distribution just after finishing DXR administration

**FIGURE 2:**
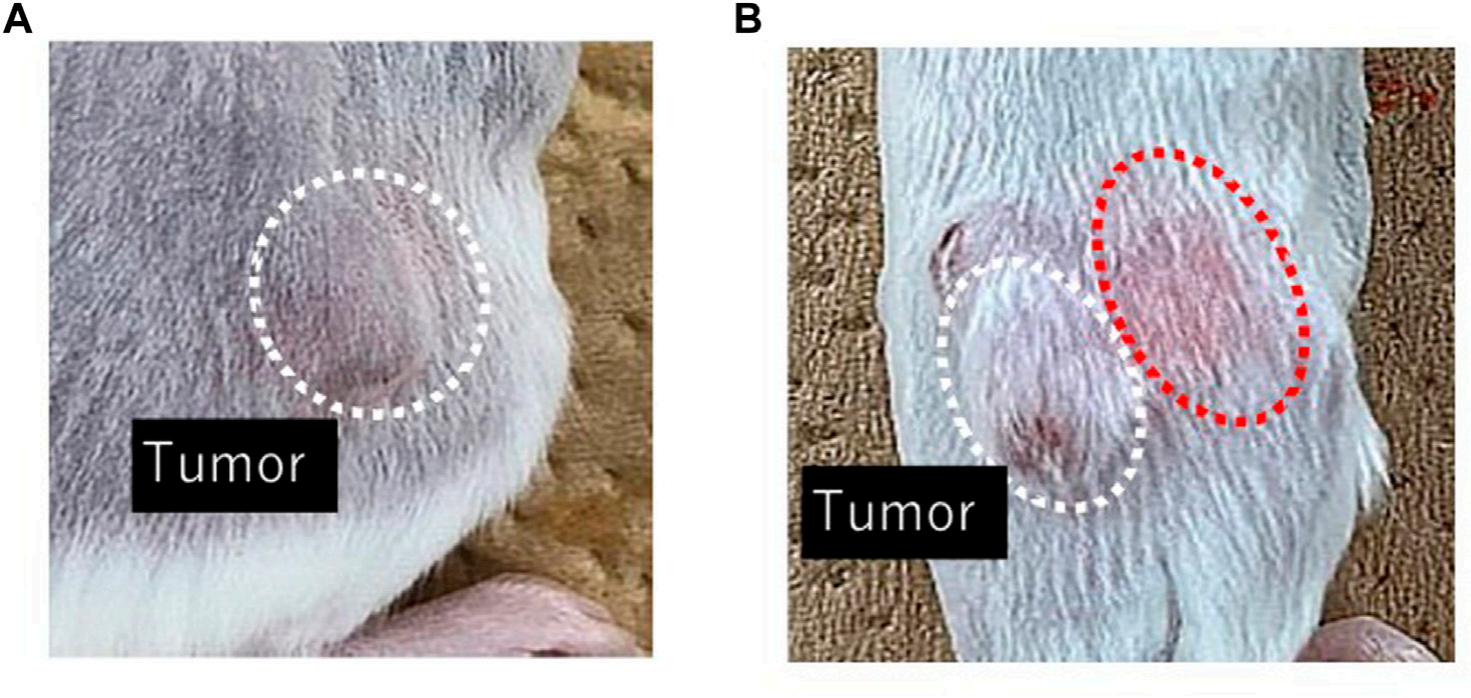
Visible observation of DXR distribution just after finishing drug administration with an EO pump **(A)** and a syringe **(B)**. Representative images of the area around a tumor in each group (*n* = 5).

Figure 2 shows DXR distribution after *i.t.* administration of DXR with a syringe and an EO pump. Rapid DXR administration was conducted with a syringe, and a red color derived from DXR was confirmed in the peripheral part of tumor tissue by visible observation, whereas no red color was observed after EO pump administration.

### Suppression of tumor growth

**FIGURE 3:**
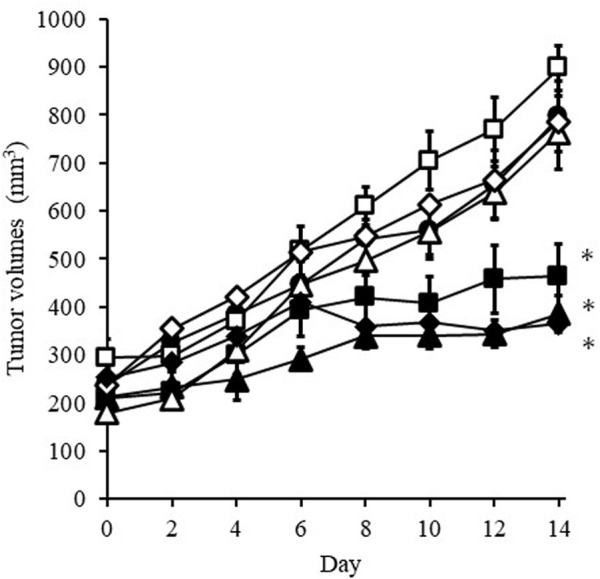
Change in tumor volume after administration of DXR solution or PBS every 2 days. Symbols; •: *i.v.* injection of PBS, ◇: *i.v*. injection of DXR at a dose of 20 μg, ■: *i.t.* administration of DXR with a syringe at a dose of 20 μg, △: iadministration of PBS with an EO pump, □: *i.t.* administration of PBS with a syringe, ▲: *i.t.* administration of DXR with an EO pump at a dose of 20 μg, ◆: *i.v.* injection of DXR at a dose of 100 µg. Each point shows the mean ± S.E. (*n* = 5). * Significant difference (*p* < 0.05) was observed compared with *i.v.* injection of PBS.

Figure 3 shows that change in the tumor volume after the administration of DXR solution or PBS every 2 days. Significant tumor growth suppression was detected after *i.v.* administration with 100 µg DXR. When bolus *i.v.* injection or rapid *i.t.* administration of PBS was conducted, the size of tumors increased independently of the administration route. The same tendency was also confirmed when *i.t.* administration of PBS was performed using an EO pump. On the other hand, *i.t.* administration of DXR at a dose of 20 µg (both after administration using an EO pump of after rapid administration with a syringe) showed a significant (*p* < 0.05) suppression of tumor growth compared with *i.v.* injection of PBS, which was also observed after *i.v*. administration of DXR at a 100 µg dose, regardless of administration rate. When *i.v.* administration of DXR at a dose of 20 µg was performed, growth of tumors tissue was apparent, as with *i.v.* injection and *i.t.* administration of PBS.

### Imaging analysis of luciferase luminescence in tumor tissue

**FIGURE 4:**
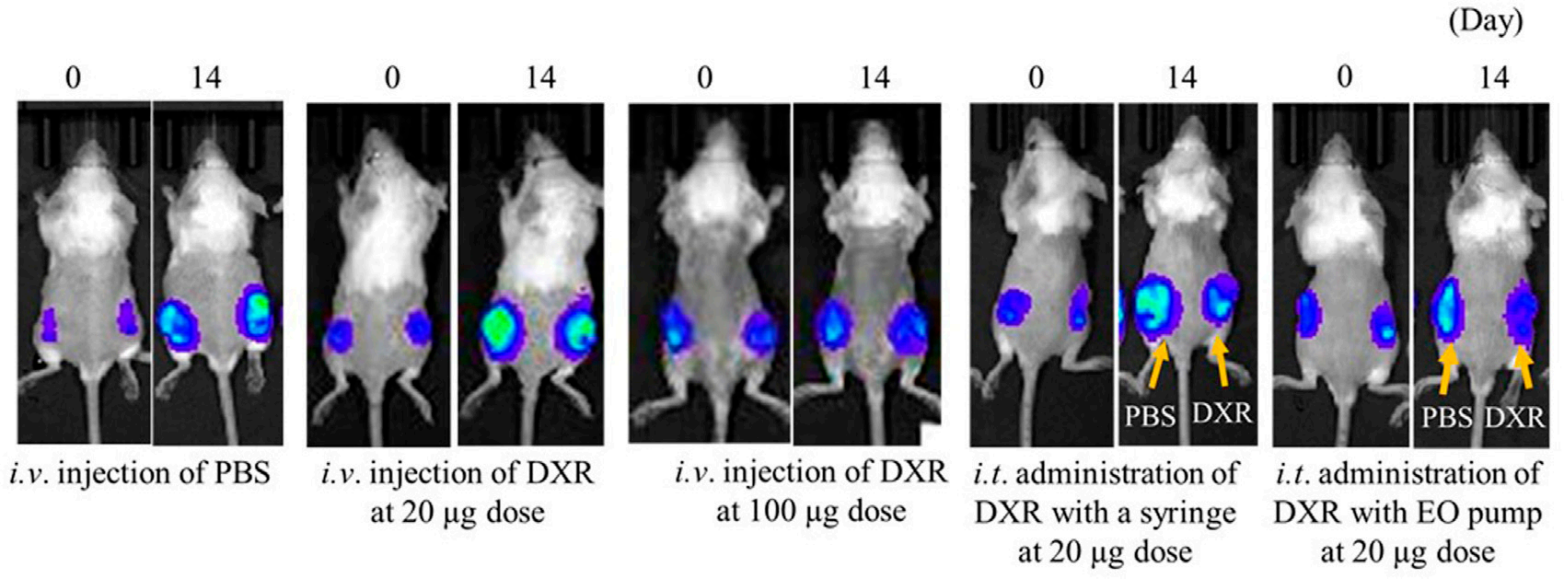
Luciferase luminescence image for subcutaneous transplanted 4T1/Luc cells in mice after 20 µg or 100 µg of DXR was administered. Representative images of tumor-bearing mice in each group (*n* = 5).

Figure 4 shows luciferase luminescence images of subcutaneously transplanted 4T1/Luc cells in mice after 20 µg or 100 µg of DXR was administered. When *i.t.* administration of DXR was done to one of the two tumor tissues with a syringe or an EO pump, PBS was injected into the other tumor tissue with the same device.

After 14 days of *i.v.* administration of low-dose DXR or PBS, an increase in luciferase luminescence was observed in tumor tissues compared with before administration. On the other hand, equal to slightly increased luciferase luminescence was confirmed after *i.v*. injection of DXR at a dose of 20 µg compared with before injection. When *i.t.* administration of DXR was conducted with a syringe and an EO pump, only slight increases in luciferase luminescence were observed compared with before the administration of DXR. Furthermore, compared with the PBS administration site, decreased luciferase luminescence was confirmed 14 days after *i.t.* administration of DXR, regardless of the administration rate.

### Change in body weight

**FIGURE 5:**
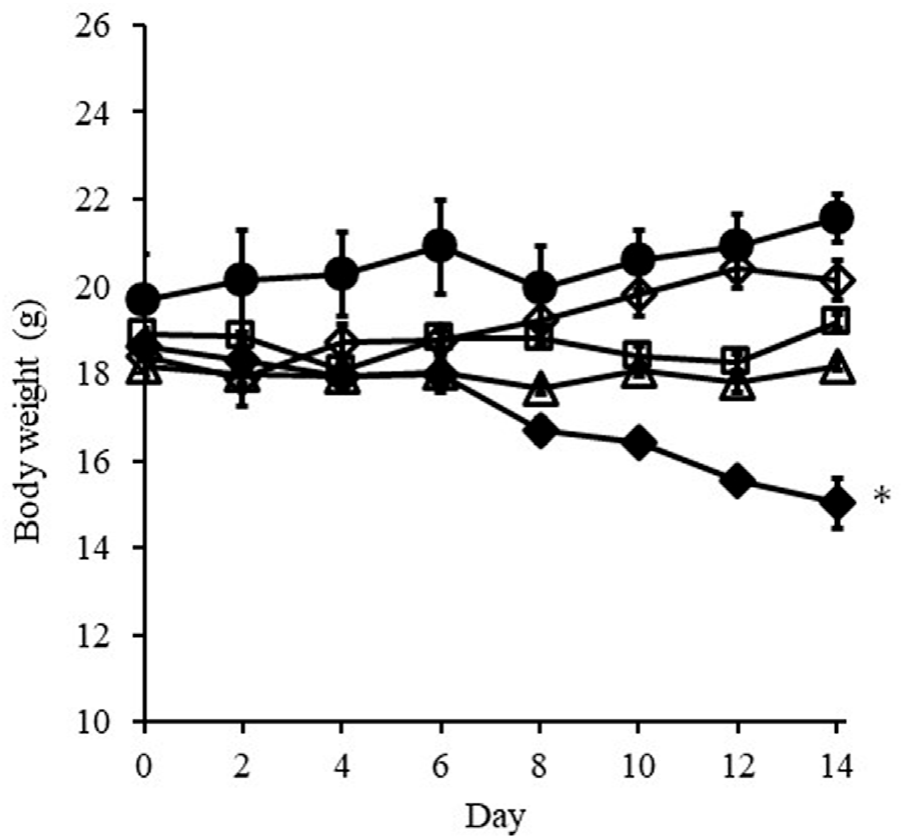
Change in body weight of mice treated with *i.v.* injection or *i.t.* administration of DXR over 14 days. Symbols; ○: *i.v.* injection of PBS, ◇: *i.v.* injection of DXR at a dose of 20 μg, □: *i.t.* administration of DXR with a syringe at a dose of 20 μg, △: *i.t.* administration of DXR with EO pump at a dose of 20 μg, ◆: *i.v.* injection of DXR at a dose of 100 µg. Each point shows the mean ± S.E. (*n* = 5). * Significant difference (*p* < 0.05) was observed compared with *i.v.* injection of PBS. White dots line shows that tumor, and the red dotted line shows the red color derived from DXR distribution.

Figure 5 shows change in the body weight of mice treated with *i.v.* injection or *i.t.* administration of DXR over 14 days. Body weight loss was observed with *i.v.* injection of DXR at a dose of 100 µg from 8 days, and significant weight loss (*p* < 0.01) was detected at 14 days after injection compared with *i.v.* injection of PBS. On the other hand, no significant body weight loss was confirmed when *i.t.* administration of DXR at a dose of 20 µg with a syringe or an EO pump. Likewise, no effect on body weight was exhibited after *i.v.* administration of DXR at a dose of 20 µg.

### Measurement of DXR in tumor, its peripheral tissue, and plasma

**FIGURE 6:**
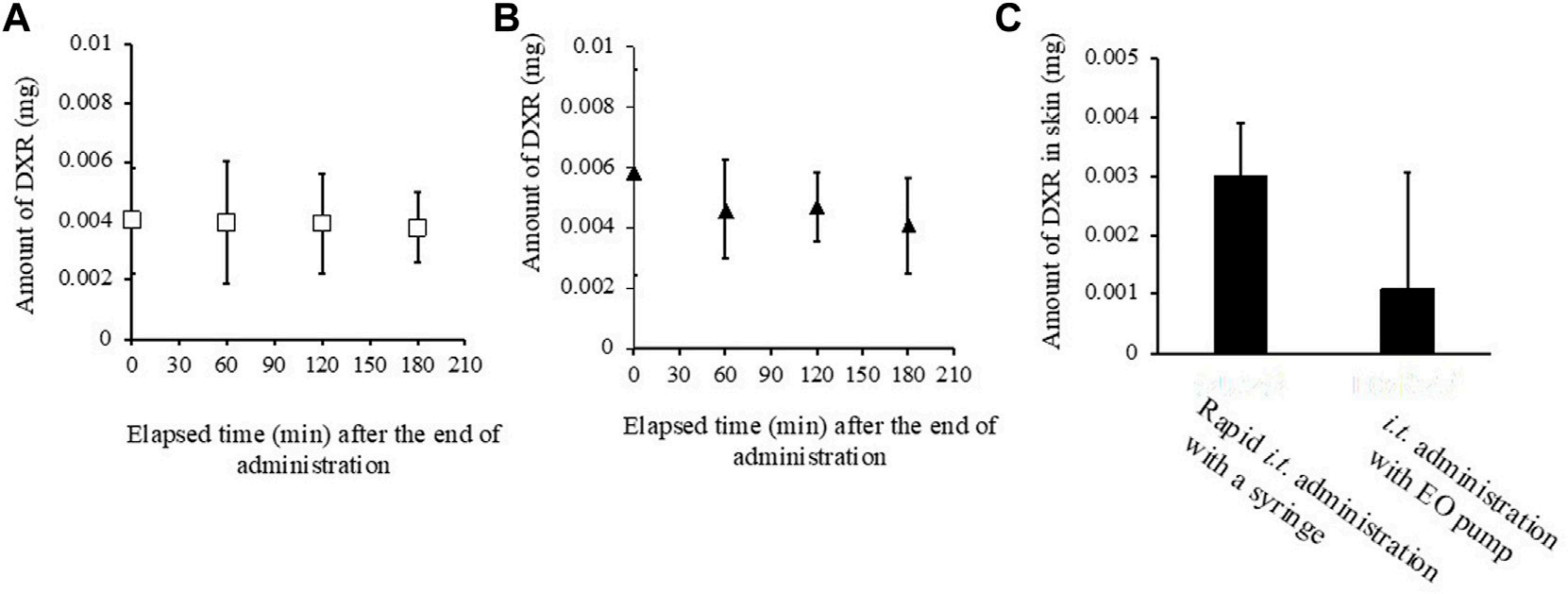
Amount of DXR in tumor **(A, B)**, its peripheral skin around the tumor site **(C)**. **(A)** rapid *i.t.* administration of DXR with a syringe at a dose of 20 μg, **(B)** *i.t.* administration of DXR with an EO pump at a dose of 20 μg, **(C)** the amount of DXR in the perirenal skin around tumor site, Each point shows the mean ± S.E. (*n* = 6).

Figures 6A, B show the change in the amount of DXR in tumors just after finishing *i.t.* administration with a syringe and an EO pump. In the case of administration using an EO pump, the amount of DXR was measured just after finishing continuous administration over about 30 min. In both cases, almost the same amount of DXR distribution was detected in the tumors just after finishing administration. The amount of DXR was then measured over a subsequent time course of 180 min, and little decrease in the amount of DXR in the tumors was observed between the two administration methods. On the other hand, when the DXR amount in tumor peripheral tissue was measured, higher amounts of DXR were detected after direct *i.t.* administration with a syringe compared with that with an EO pump (Figure 6C).

## Discussion

The blood concentration of a drug is affected by the injection rate. The steady state of blood concentration (C_ss_) after *i.v.* infusion can be expressed using the following equation (Johan and Daniel, 1997):

$$C_{ss}=infusion\;rate/drug\;clearance$$

Because drug clearance is associated with the plasma half-life of an infused drug, direct administration of anticancer drugs into tumor tissues at a slow rate results in lower blood concentration and is considered as a treatment approach to reduce side effects.

Direct *i.t*. administration of anticancer drugs has been reported with thermally sensitive formulations and gel formulations (Aquib et al., 2020). Tumor treatment with these implantable formulations may be more acceptable compared with invasive surgical treatment, although it requires the drug to be retained in the body for an extended period. On the other hand, the Alzet mini-osmotic pump which can direct intratumoral drug delivery has been reported (Wang et al., 2018). The growth of glioma tumor implanted with the pump was effectively suppressed, but flow rate cannot be controlled. Supplementary Figure S1 show that the flow rate is increased in proportion to the increase in voltage by EO pump used in this study. Using drug administration with an EO pump used in this study, it will be possible to change detailed dosage modification according to symptoms in case of side effects caused by anti-cancer drugs.

Khan et al. (2005) evaluated the distribution of transferrin-doxorubicin complexes in brain tumors by injecting them into normal or tumor tissues at different injection rates. They reported that although there was no effect of injection rate on doxorubicin distribution in the brain when transferrin-doxorubicin complexes were administered into normal tissues, the distribution was approximately 3-fold higher when the complexes were injected into tumor tissues at a slower rate.

In the present study, DXR administration with the EO pump showed the therapeutic effect of DXR without decreasing body weight. Although further study is needed, it is possible that the distribution of DXR in the normal tissue adjacent to the tumor tissue and the blood concentration may be different from its rapid administration.

It has been reported that drug distribution at the site was affected by the drug administration rate. Kim et al. (2017) reported that fast injection of a drug into subcutaneous tissue widely permeates in a horizontal direction more than in a vertical direction by forming depots in an elliptical shape. This was attributed to the diffusion pattern of the injected solution being largely influenced by mechanical prosperities of the tissue. Therefore, a slowly injected drug solution may spread less than when administered rapidly. In the present study, a higher amount of DXR and a red color derived from DXR were observed in peripheral skin tissue around the tumor when DXR was administered rapidly with a syringe. However, no significant difference was observed in the amount of DXR in the tumor with either slower administration with an EO pump or using rapid administration with a syringe. This result also revealed that slower injection with an EO pump may be better to minimized side effects caused by the administrated drug. Further investigation will be necessary to show the usefulness of administering anticancer drugs at lower speeds, but because there are limitations to manually injecting at a slower rate, an EO pump-based administration method seems to have the potential and is feasible.

## Conclusion

When anticancer drug DXR was administered at lower administration rate (0.6 µL/min) with an EO pump, high therapeutic efficacy without any side effects such as weight loss was observed. In addition, the blood concentration and the peripheral skin concentration of DXR after administration at lower rate with EO pump were decreased compared with those after the rapidly administration with a syringe.

In addition to conventional anticancer drug therapy, local drug administration therapy using an EO pump, which can be administered at lower administration rate that are difficult to administer manually, may lead to a wider range of treatment options for patients. Furthermore, even drug candidates that have been disregarded because of poor selectivity to target tissues may be able to resume development by utilization of an EO pump. Based on the above, EO pump-based drug administration methods may be useful for local therapy with anticancer drugs. However, in the present study, anticancer effect provided by EO pump was confirmed by every 2 days administration of DXR. Thus, further study of modification of the dosage and administration rate would be necessary to implement this method in practical uses.

## Data Availability

The raw data supporting the conclusion of this article will be made available by the authors, without undue reservation.
