# Supplementary material for: Usefulness of direct intratumoral administration of doxorubicin hydrochloride with an electro-osmosis–assisted pump
Source: Front Drug Deliv. 2023 Apr 4;3:1150894. doi: 10.3389/fddev.2023.1150894 (PMC12363246; doi:10.3389/fddev.2023.1150894)
Supplement: Supplementary file 1 [file DataSheet1.PDF]

Supplementary Information

Usefulness of direct intratumoral administration of doxorubicin hydrochloride with an electro-osmosis–assisted pump

Ayu Ito[1], Shoko Itakura[1], Yuya Hasegawa[1], Miyu Hashimoto[1], Akie Okada[1], Mamoru Hirafuji[2], Hidenori Nakamura[2], Kenji Sugibayashi[1,3], Hiroaki Todo[1,*].

[1] Faculty of Pharmacy and Pharmaceutical Sciences, Josai University, 1-1 Keyakidai, Sakado, Saitama 350-0295, Japan.

[2] atDose Co., Ltd. 2-41-54 Tsurugamine, Asahi-ku, Yokohama, Kanagawa 241-0022, Japan.

[3] Faculty of Pharmaceutical Sciences, Josai International University, 1 Gumyo, Togane, Chiba-ken 283-8555, Japan

*** Correspondence:**
Hiroaki Todo
ht-todo@josai.ac.jp

Supplemental Figure 1.

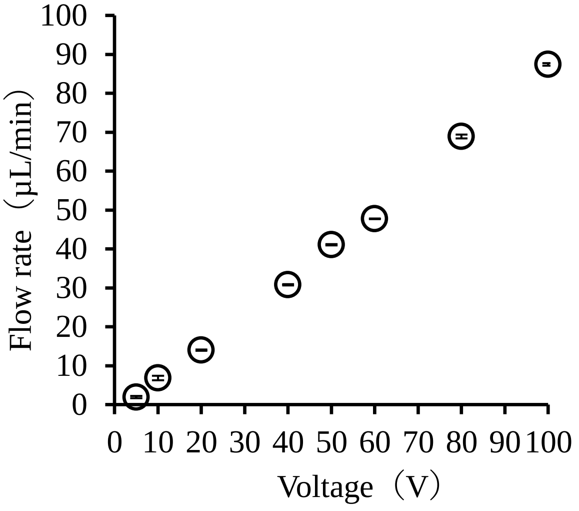

Supplemental Figure 1. The relationship between applied voltage and flow rate.
Each point shows the mean ± S.D. (n = 5).
